# Online video versus face-to-face patient–surgeon consultation: a systematic review

**DOI:** 10.1007/s00464-024-11307-7

**Published:** 2024-11-05

**Authors:** Britte H. E. A. ten Haaft, Roberto M. Montorsi, Esther Barsom, Geert Kazemier, Marlies P. Schijven, Marc G. Besselink

**Affiliations:** 1 Department of Surgery, Amsterdam UMC, Location University of Amsterdam, Amsterdam, The Netherlands; 2 Cancer Center Amsterdam, Amsterdam, The Netherlands; 3 Department of General and Pancreatic Surgery, The Pancreas Institute, University of Verona Hospital Trust, Verona, Italy; 4 Department of Surgery, Amsterdam UMC, Location Vrije Universiteit, Amsterdam, The Netherlands; 5 Amsterdam Public Health, Digital Health, Amsterdam, The Netherlands; 6 Amsterdam Gastroenterology Endocrinology Metabolism, Amsterdam, The Netherlands

**Keywords:** Online video consultation, Face-to-face consultation, Surgery, Satisfaction, Efficacy, Information recall

## Abstract

**Background:**

Online video consultation (OVC) is increasingly used in patient–surgeon pre-surgical and follow-up consultation but a comprehensive review assessing its benefits and downsides as compared to face-to-face (F2F) consultation is currently lacking. This systematic review evaluated the effectiveness of OVC as compared to F2F consultation.

**Methods:**

A literature search (Ovid/Medline, Embase, and Clarivate Analytics/Web of Science Core Collection) was conducted including studies comparing efficacy, patient and surgeon satisfaction, and information recall between OVC and F2F patient–surgeon consultation (inception-December 4, 2023).

**Results:**

Out of 1021 studies, 14 studies with 13,564 patients met the eligibility criteria, consisting of seven RCTs, three prospective, and four retrospective studies. Various types of surgical consultations were evaluated, including new referrals, routine follow-ups, postoperative follow-ups, and mixed consultations (both pre- and postoperative). None of the randomized trials exclusively compared OVC with F2F consultations in the high-demand preoperative setting, or assessed patient information recall. Efficacy outcomes were reported by seven studies. Among these, three RCTs showed that OVC improved efficacy in terms of waiting time (8.2 vs. 20.7 min, *P* = 0.01) and total appointment time (24 vs 71 min, *P* = 0.001, and 21.9 vs. 154.8 min, *P* = 0.001). Patient satisfaction was reported by 10 studies. Regarding patient satisfaction, one “mixed design” study favoured OVC (92% vs. 63%, *P* = 0.04), while eight studies reported similar outcomes.

**Conclusions:**

This systematic review identified some benefits of OVC such as shorter waiting and total appointment times as compared to F2F patient–surgeon consultation, although the true value of OVC remains unknown due to the limited available evidence. Future pragmatic RCTs are needed, which should include the pre-surgical consultation and assess patient information recall.

**Supplementary Information:**

The online version contains supplementary material available at 10.1007/s00464-024-11307-7.

Traditionally, patient–surgeon consultations have been performed face to face (F2F), with telephone consultations (TC) being used in specific settings, such as follow-up for select indications. In recent years, and specifically during the COVID-19 pandemic, this changed and online video consultation (OVC) strategies were rapidly implemented in many hospitals worldwide to maintain access to essential surgical care [1]. OVC offers unique advantages over TC by enabling verbal communication between patients and surgeons, while preserving non-verbal communication [2].

After the COVID-19 pandemic, OVC could continue to play a meaningful and increasing role by ensuring healthcare accessibility, particularly in the context of the growing centralization of healthcare systems which has increased travel distances [3]. Herein, especially patients with difficulties to travel and those at large distances from caregivers could benefit from OVC. In addition, OVC may have a positive impact on economic and environmental aspects by reducing travel-related expenses and healthcare-related carbon footprint [4]. During recent years, different OVC strategies have shown their feasibility across various settings [5]. A comprehensive understanding of associated barriers of OVC is crucial to continue its implementation. These barriers encompass the inability to conduct hands-on physical examinations, digital literacy challenges, and issues related to health literacy. The balance between benefits and barriers may vary across specialties. This balance might be even more delicate in surgical care, given the required technical explanation and consent for surgical procedures [6]. However, the specific benefits and limitations of OVC as compared to F2F are poorly studied in the surgical setting, and a systematic review is, thus far, lacking.

We performed this systematic review to assess the value of OVC compared to F2F patient–surgeon pre-surgical and follow-up consultations, focusing on levels of satisfaction for both patients and surgeons, efficacy, and patient information recall.

## Methods

The conduct and reporting of this review adhere to the Preferred Reporting Items for Systematic Reviews and Meta-Analyses (PRISMA)-statement [7, 8]. This review was registered in PROSPERO under registration number CRD42023399954.

### Search strategy

After several scoping searches, three bibliographic databases (Ovid/Medline through PubMed, Embase, and Clarivate Analytics/Web of Science Core Collection) were searched for relevant literature from inception to December 4, 2023. Searches were devised in collaboration with a medical information specialist (KAZ). Search terms including synonyms, closely related words, and keywords were used as index terms or free-text words: “video consultation” and “surgery”. The searches contained no methodological search filter, date, or language restrictions that would limit results to specific study type, date, and language. Duplicate articles were excluded using AmcDedupEndnote, a java script developed by Geert Lobbestael (version 0.9.6), and a manual check in EndNote (v20) by KAZ. The full search strategy used for each database is detailed in Supplementary Material—File 1.

### Selection process

Two reviewers (BTH and RMM) independently screened all potentially relevant titles and abstracts for eligibility using Rayyan (version 2022) [9]. If necessary, the full-text article was checked for the eligibility criteria. Differences in judgement were resolved through a consensus procedure and, if necessary, with the opinion of a third reviewer (MGB). Studies were included if they met the following criteria: (i) surgical patients (patients who had an appointment with a surgical healthcare provider) **(P)**; (ii) receiving online video consultation **(I)**; (iii) compared to face-to-face consultation **(C)**; (iv) investigating patient and surgeon satisfaction, information recall, environmental impact, efficacy (i.e. appointment time, travel time, costs) **(O)**; (v) published from inception to December 4, 2023 **(T);** (vi) randomized controlled trials (RCTs), and prospective and retrospective studies. We excluded studies if they were (i) trial protocols, conference abstracts, secondary publications of previously published studies, commentaries, and articles without an available full text; (ii) publications in languages other than English. Data extraction included: publication details (i.e. study title, publication date, authors, study design), baseline characteristics (i.e. number of patients, sex, age, diagnosis and if available digital and overall literacy), and intervention characteristics (i.e. video consultation platform, duration of consultation, and technical difficulties). Primary and secondary endpoints were extracted.

### Critical appraisal

Two reviewers (BTH and RMM) independently evaluated the methodological quality of the full-text papers using the Cochrane Risk of Bias 2 (RoB-2 [10]) tool for RCTs. The risk of bias in non-randomized studies (ROBINS-1 [11]) tool was used for all studies other than RCTs. Disagreements were resolved by consensus and, if necessary, by the opinion of a third reviewer (MGB). We adjudicated risk of bias as low only if all domains were assessed as low risk of bias. The overall risk-of-bias judgement is shown in Table 1. A complete overview of the critical appraisal is displayed in Supplementary Material—File 2—Table 1 and Supplementary Material—File 3—Table 2.

**Table 1:** Characteristics of included studies on online video patient–surgeon consultation

Author (year)	Country	Type of surgery	Type of consultation	Study design	No. of patients (OVC;F2F)	Age	Female (%)	Primary outcome	Overall risk-of-bias judgement
Westra (2015)	NL	Plastic surgery	Postop FU	RCT	31 (16;15)	OVC: 46.25 (9.60) F2F: 55.47 (11.60)	25 (80.6)	Patient satisfaction	Some concerns
Viers (2015)	USA	Urology	Postop FU	RCT	55 (28; 27)	62.0 (8.1)	NR	Efficacy	Some concerns
Buvik (2016)	NO	Orthopedic	New referrals, regular FU, mixed (both pre- postop) consultation	RCT	389 (199; 190)	OVC: 45% (19y-64y) F2F: 45% (19y-64y)	241 (60.6)	Surgeon satisfaction	Some concerns
Sellars (2020)	UK	Colorectal surgery	New referrals	PC	281 (50;231)	OVC 68 [36–90] F2F: 69 [17–90]	160 (56.9)	Efficacy	Serious risk
Damery (2021)	UK	Liver transplant	Postop FU	RCT	54 (29; 25)	48.9 (13.8)	22 (41)	Patient satisfaction	High risk
Lee (2021)	USA	Urogynecology	Postop FU	RCT	52 (26; 26)	OVC: 59.9 (10.9) F2F: 58.0 (11.3)	52 (100)	Patient satisfaction	Some concerns
Barsom (2021)	NL	Colorectal surgery	Postop FU	PC	50 (21; 29)	OVC 61 [53–69] F2F: 68 [57–74]	28 (56)	Patient satisfaction	Serious risk
Schumm (2021)	USA	Endocrine surgery	Postop FU	PC	77 (45; 32)	OVC: 46 [39–57] F2F: 59 [43–64]	58 (75.3)	Patient satisfaction	Serious risk
Sharma (2021)	USA	Neurosurgery	Regular FU	RC	9375^A^ (4571; 4804)	NR	NR	Patient satisfaction	Critical risk
Sibanda (2021)	UK	Trauma surgery	New referrals and FU consultation	RC	54 (24; 30)	NR	NR	Efficacy	Serious risk
Muschol (2022)	GER	Orthopedic and trauma surgery	FU consultation	RCT	52 (26; 26)	OVC: 65% (41y-60y) F2F: 58% (41y-60y)	21 (40.4)	Efficacy	Some concerns
Mahmoud (2022)	EG	Pediatric surgery	Mixed (both pre- postop) consultation	MC	2268 (872; 1056)	OVC: 1.2 (3.6) F2F: 1.6 (2.7)	905 (39.9)	Patient satisfaction	Serious risk
Baxter (2023)	USA	Orthopedic	Preop consultation	RC	802 (459; 343)	OVC: 69 [35–93] F2F: 70 [32–91]	F2F: 40.2 OVC: 35.1	Efficacy	Serious risk
Sada (2023)	USA	Bariatric surgery	Postop FU	RCT	24 (13; 11)	OVC: 48.7 (11.9) F2F: 50.3 (10.5)	15 (62.5)	Patient satisfaction	High risk

Results are expressed as means (SD) or medians [IQR]; Synonyms of Face-2-Face (F2F) consultation or online video consultation (OVC) are expressed as F2F or OVC accordingly

*NL* The Netherlands, *USA* United States of America, *UK* United Kingdom, *NO* Norway, *GE* Germany, *EG* Egypt, *postop* postoperative, *preop* preoperative, *RCT* randomized controlled trial, *PC* prospective cohort study, *RC* retrospective cohort study, *MC* mixed (prospective and retrospective) cohort study, *NR* not reported, *FU* follow-up

^A^Number of consultation

**Table 2:** Efficacy of online video patient–surgeon consultation

Author (year)	Number of patients (OVC;F2F)	Study design	Efficacy assessment	Online video consultation Mean (SD)/median [IQR] / %	F2F consultation Mean (SD)/median [IQR] / %	*P* value
Westra (2015)	31 (16;15)	RCT	Waiting time (min)	8.1 (9.8)	20.7 (16.3)	0.01
Viers (2015)	55 (28; 27)	RCT	Waiting time (min)	12.5 (14.4)	16.4 (15.4)	0.27
Sellars (2020)	281 (50; 231)	PC	Travel time (saved) (min)	240 [8–390]	46 [6–330]	NR
Lee (2021)	52 (26; 26)	RCT	Duration of visit (min)	24 (5.8)	71 (22)	0.001
Sibanda (2021)	54 (24; 30)	RC	ACLS (validated, range 0–8)	7.8	8.0	0.33
Muschol (2022)	52 (26; 26)	RCT	Total time appointment (min)	21.9 (10.4)	154.8 (79.8)	0.001
Baxter (2023)	802 (459; 343)	RC	30-day readmission	21 (4.6)	14 (4.1)	0.735

Results are expressed as means [SD] or medians (IQR); Synonyms of Face-2-Face (F2F) consultation or online video consultation (OVC) are expressed as F2F or OVC accordingly

*RCT* randomized controlled trial, *PC* prospective cohort study, *RC* retrospective cohort study, *NR* not reported, *ACLS* Ashford Clinic Letter Scoring System

### Statistical analysis

Descriptive statistics were used to summarize the extracted data. Continuous data were presented as mean with standard deviation (SD) or as median with interquartile range [IQR]. Binary or categorical data were presented as frequencies with percentages. We interpreted two-sided *P* < 0.05 as statistically significant. Due to the heterogeneity of clinical features, study design, and outcome assessments, especially due to the use of different (validated) questionnaires, a meta-analysis was considered not feasible.

## Results

### Search results

**Fig. 1:**
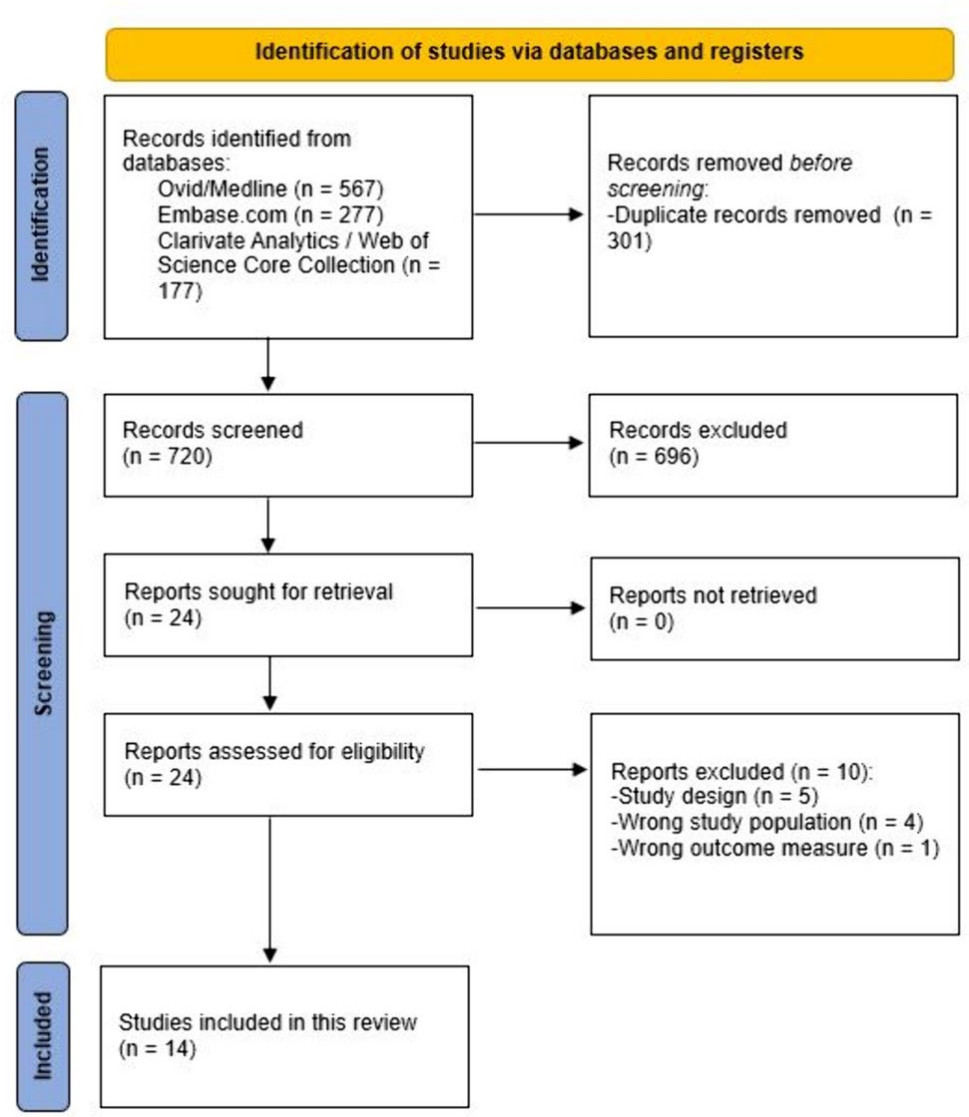
Flowchart of the search and selection procedure of studies

The literature search generated 1021 references: 567 in Ovid/Medline, 277 in Embase, and 177 in Clarivate Analytics/Web of Science Core Collection. After removing duplicates of references from more than one database, 720 references remained. The flow chart of the search and selection process is presented in Fig. 1.

### Characteristics of identified studies

Overall, 14 eligible studies with 13,564 patients could be included [4, 12–24]. Study characteristics are presented in Table 1. The included studies were performed in the United States (*n* = 6) [13, 17, 19, 20, 23, 24], United Kingdom (*n* = 3) [15, 16, 21], the Netherlands (*n* = 2) [12, 18], Germany (*n* = 1) [4], Egypt (*n* = 1) [22], and Norway (*n* = 1) [14]. Diverse methodologies were employed, including seven RCTs [4, 12–14, 16, 17, 24], three prospective cohort studies [15, 18, 19], three retrospective cohort studies [20, 21, 23], and one “mixed design” prospective-retrospective cohort study [22]. Various types of surgical consultations were evaluated, including new referrals, routine follow-ups, postoperative follow-ups, and mixed consultations (both pre- and postoperative). Seven of the 14 studies assessed OVC during postoperative follow-up consultation whereas the other seven studied new referrals, routine follow-ups, or mixed consultations (both pre- and postoperative). None of the randomized trials exclusively compared OVC with F2F consultations in the high-demand preoperative setting involving detailed explanations of surgical procedures and patient-informed consent.

The research was conducted across eight different surgical specialisms with two prospective studies specifically performed in surgical oncology departments. Cohorts ranged from 24 to 2268 patients, with a median age ranging from 46 to 70 years (excluding the paediatric study with an age range of 1.2–1.6 years). Both men and women were included. The reported outcomes included patient satisfaction (*n* = 9), surgeon satisfaction (*n* = 4), and the efficacy of OVC, encompassing consultation duration, waiting time, and travel time (*n* = 7). No study reported on patient information recall.

### Efficacy

The efficacy of OVC compared to F2F consultation was assessed in seven studies (including 4 RCTs), see Table 2. One of two RCTs which assessed waiting time demonstrated a significant reduction in waiting time for OVC (8.1 vs 20.7 min, *P* = 0.01) [12] while a second RCT in 55 patients found no significant difference in waiting time (12.5 vs 16.4 min, *P* = 0.27) between OVC and F2F, respectively [13]. Both RCTs which assessed total appointment time (with and without travel time) showed significant time benefits with OVC (when excluding travel time: 24 vs 71 min, *P* = 0.001 and when including travel time: 21.9 vs 154.8 min, *P* = 0.001) [4, 17]. Regarding 30-day readmission rates, one 2023 American retrospective study found no significant differences (21% vs 14%, *P* = 0.735) [23].

### Patient satisfaction

Patient satisfaction was assessed in nine studies (including 5 RCTs), employing seven different satisfaction questionnaires, with three of them being validated instruments: Patient Satisfaction Questionnaire (PSQ-18), Visit-Specific Satisfaction Instrument (VSQ-9), and CAHPS (Consumer Assessment of Healthcare Providers and Systems) [25–27]. Among these nine studies, eight found no significant difference in patient satisfaction between OVC and F2F consultation (Table 3). One 2015 Dutch RCT, enrolling 31 patients after plastic surgery, reported comparable satisfaction rates between OVC and F2F consultation [12]. Subgroup analyses revealed a higher general satisfaction after OVC (3.91 [IQR: 0.55] vs 3.57 [IQR: 0.65], *P* = 0.02), as well as for accessibility (3.58 [IQR: 0.45] vs 4.10 [IQR: 0.62], *P* = 0.01), as compared to F2F consultation. Other studies expressed patient satisfaction as percentages, ranging from 80 to 96% for OVC versus 65 to 94% for F2F [16–20]. Conversely, a 2022 Egyptian study in 2268 patients after paediatric surgery reported a higher patient satisfaction by both parents and surgeons in the OVC group (92% vs 63%, *P* = 0.04). This “mixed design” prospective-retrospective study utilized a non-validated Patient Experience Assessment (PEA) comprising 22 questions, commonly employed as part of their standard hospital policy to enhance service quality [22].

**Table 3:** Patient satisfaction of online video patient–surgeon consultation

Author (year)	Number of patients (OVC;F2F)	Study design	Satisfaction assessment	Online video consultation Mean (SD)/median [IQR] /%	F2F consultation Mean (SD)/median [IQR] /%	*P* value
Westra (2015)	31 (16;15)	RCT	PSQ-18 (validated, 5-point likert scale, 1 = strongly disagree, range 1–5)	3.79 (0.40)	3.97 (0.46)	0.35
Viers (2015)	55 (28; 27)	RCT	One question (not validated, 7-point likert scale, 1 = strongly agree, range 1–7)	1.2 (0.5)	1.1 (0.3)	0.70
Damery (2021)	54 (29; 25)	RCT	6 domains of VSQ-9 (validated, 5-point likert scale, 1 = poor, range 0–100)	80.9 (15.6)	72.7 (19.6)	0.10
Lee (2021)	52 (26; 26)	RCT	PSQ-18 (validated, 5-point likert scale, 1 = strongly disagree, range 18–90)	80.7 (2.6)	81.2 (2.8)	0.50
Barsom (2021)	50 (21; 29)	PC	PAT-VC (not validated study-specific questionnaire, 5-point likert scale, 1 = totally agree, range 0–100)	86%	65%	0.554
Schumm (2021)	77 (45; 32)	PC	CAHPS (validated, 5-point likert scale, 1 = very unsatisfied, % satisfied or higher)	96%	94%	NR
Sharma (2021)	9375^A^ (4571; 4804)	RC	CAHPS (validated, 5-point likert scale, 1 = very unsatisfied, % satisfied or higher)	85.2%	84.5%	0.307
Mahmoud (2022)	1396 (340; 1056)	MC	PEA form (not validated, 5-point likert scale, 1 = very unlikely, range 0–100)	92%	63%	0.04
Sada (2023)	24 (13; 11)	RCT	Survey of 24 questions (not validated, 5-point likert scale, 1 = very likely, number (%))	82%^B^	9%^C^	NR

Results are expressed as means [SD] or medians (IQR); Synonyms of Face-2-Face (F2F) consultation or online video consultation (OVC) are expressed as F2F or OVC accordingly

*RCT* randomized controlled trial, *PC* prospective cohort study, *RC* retrospective cohort study, *MC* mixed (prospective and retrospective) cohort study, *PSQ-18* patient satisfaction questionnaire, *VSQ-9* visit-specific questionnaire, *PAT-VC* patient video consultation, *CAHPS* consumer assessment of healthcare providers and systems, *NR* not reported, *PEA* patient experience assessment

^A^Number of consultations

^B^Percentage of group that expressed their OVC was better or as good as F2F

^C^Percentage of group that expressed OVC was worse than F2F

### Surgeon satisfaction

The four studies (including three RCTs) that assessed surgeon satisfaction are displayed in Table 4 [12–14, 18]. All studies used different assessments of which only one 2015 RCT from the Netherlands used a validated questionnaire (patient-physician experience questionnaire (PEQ)) [2, 28]. The authors reported no significant differences in surgeon satisfaction (4.19 vs 4.70, *P* = 0.09) on a 5-point Likert scale [12]. A 2021 prospective study in 50 patients after colorectal surgery from the Netherlands using a 10-item questionnaire also reported no significant differences (9.0 [8.0–9.0] vs 8.0 [7.0–8.0]) [18]. A 2021 non-inferiority RCT from Norway in 389 patients during orthopaedic outpatient visits, reported a difference in sum score of 0.1 on a 1 to 5 specialist evaluation score (1.82 vs 1.72, *P* = 0.003), well within the pre-defined non-inferiority margin. Also after subgroup analysis of type of consultation (new referrals vs. follow-up consultations), non-inferiority remained [14].

**Table 4:** Surgeon satisfaction of online video patient–surgeon consultation

Author (year)	Number of patients (OVC;F2F)	Study design	Satisfaction assessment	Online video consultation Mean (SD)/median [IQR]/%	F2F consultation Mean (SD)/median [IQR]/%	*P* value
Westra (2015)	31 (16;15)	RCT	PEQ (validated, 5-point likert scale, 1 = strongly disagree)	4.19 (0.85)	4.70 (0.24)	0.09
Viers (2015)	55 (28; 27)	RCT	Percentage of strongly agree (not validated, 0–100)	88%	90%	NR
Buvik (2016)	389 (199; 190)	RCT	Five-level questionnaire (not validated, 0–5)	1.82 (0.38)	1.72 (0.38)	0.003
Barsom (2021)	50 (21; 29)	PC	10-item questionnaire (not validated, predominantly 5-point likert scale, range 0–10)	9 [9, 10]	8 [7, 8]	NR

Results are expressed as means [SD] or medians (IQR); Synonyms of Face-2-Face (F2F) consultation or online video consultation (OVC) are expressed as F2F or OVC accordingly

*RCT* randomized controlled trial, *PC* prospective cohort study, *NR* not reported

## Discussion

This first systematic review comparing the efficacy, patient and surgeon satisfaction, and information recall between OVC and F2F patient–surgeon consultation included 14 studies, of which three RCTs, revealed shorter travel and waiting time with OVC [4, 12, 17]. Overall, patient and surgeon satisfaction levels were largely comparable between OVC and F2F consultation although there was a lack of high-quality evidence. None of the randomized trials exclusively compared OVC with F2F consultations in the high-demand preoperative setting, or assessed patient information recall.

Does OVC hold specific benefits for patients and surgeons? This study identified some benefits, namely shorter waiting time (8.1 vs 20.7 min [12]), shorter duration of visit (24 vs 71 min [17]), and shorter total appointment time (21.9 vs. 154.8 min [4]). Naturally, as OVC typically occurs from the patients’ home, it eliminates travel time. This is consistent with several studies evaluating the effectiveness of telemedicine. In an implementation study, 99% (71 out of 72) of patients agreed that OVC saved them time, while 96% (69 out of 72) stated that it also saved them money [29]. However, evaluating efficiency encompasses more than just cost and time savings; it involves other factors including appointment frequency. For instance, if a surgeon, based on the medical history or current complaints, opts to conduct additional physical exams or lab tests, this could result in extra in-person appointments when initially using OVC. Conversely, such procedures can be easily integrated after a F2F consultation. Hence, a comprehensive exploration of its overall efficiency is essential.

The present review found rather consistent results regarding patient and surgeon satisfaction between OVC and F2F consultation. This aligns with the satisfaction rates reported with OVC in primary healthcare. A recent scoping review focusing on OVC in 13 studies in primary healthcare reported that 94–99% of patients were “very satisfied” with OVC, citing reduced travel times, no waiting time in waiting rooms, and improved access to general practioners [30]. Also, studies in anaesthesiology, gynaecology, and oncology demonstrated positive attitudes towards OVC among patients. A 2022 single-centre study surveyed 2805 patients on their experiences with a preoperative anaesthetic OVC, and reported 100% agreement (72 out of 72 patients) regarding clarity of anaesthetic explanations and feeling listened to. A minority (19%) of patients expressed a preference for F2F consultation, primarily due to limited prior experience with video platforms and concerns about limited airway assessments [29]. A 2021 survey among 53 patients after OVC in gynaecological oncology reported an overall satisfaction score of 90.5% [31]. Finally, a recently published RCT evaluated the effect of online video versus F2F palliative care on the quality of life of 1250 patients with advanced lung disease, and reported equivalent outcomes [32]. Additionally, subgroup analysis of type of consultation (new referrals vs follow-up consultations) performed by Buvik et al. 2016 [14], reported no statistically significant differences among OVC and F2F. Despite the limited number of studies investigating the type of consultation, these results strengthen the concept that surgical outcomes do not seem to be affected by the type of consultation, leading the way for future high-quality studies on this topic.

In the present study, satisfaction levels for OVC and F2F consultation also appeared largely similar between surgeons. However, only one RCT utilized a validated assessment tool to report satisfaction rates [12]. Similarly, in a 2021 survey among 109 physicians from family medicine, over 90% expressed either high or moderate satisfaction with OVC [33]. This finding has been also been reported in specialties such as psychiatry and anaesthesia [34, 35]. In a 2023 online survey, 73.8% of 145 responding physicians from various departments at a university hospital expressed satisfaction with teleconsultation (including OVC and TC), with 79.3% believing in its continued use in the future [35].

The increasing adoption of OVC since COVID-19 could present challenges for patients less familiar with technology, potentially adding stress to an already stressful situation [36]. However, for computer literate individuals, OVC seems to offers benefits like time and travel savings, especially crucial in today's centralized care model, but formal assessments are rare. In addition, considering the urgency of global warming, exploring how OVC can contribute to reducing the healthcare carbon footprint is of importance [37]. Future studies should carefully choose their methodology in this respect [38].

The results of this systematic review must be interpreted considering several limitations. First, a substantial heterogeneity in methods of assessment among studies was observed which makes it challenging to draw definitive conclusions. Among the three validated patient satisfaction questionnaires, PSQ-18 featured in two RCTs, assessing seven domains [25]. The other two validated questionnaires, PEQ and CAHPS, predominantly concentrate on surgeon skills [27, 28]. While the patient–surgeon relationship is vital, the satisfaction evaluation conducted by PSQ-18 encompasses a broader spectrum compared to PEQ and CAHPS, thus, providing a more comprehensive reflection of satisfaction. Second, it is important to note that many of the papers were rated as having a serious or high risk of bias. Third, the observed reduction in time may result from differences in the processes before the appointment, including registration and check-out procedures. However, this also reflects standard practice and can be seen as an advantage of OVC, thereby generally making the process less time consuming. Fourth, this study solely focused on patients' efficacy in terms of time and costs. To fully evaluate efficacy outcomes, future studies should also consider efficacy from the perspectives of healthcare providers and hospitals. Moreover, the actual efficacy outcomes largely depend on the calculation method used (e.g. whether travel time, registration time, etc., were included or excluded), which should be carefully considered when interpreting these results. Fifth, this review included a variety of surgical subspecialties, encompassing eight different surgical specialties rather than focusing on one specific surgical department. However, the inclusion of various subspecialties contributes to the external validity of the study. Additionally, age, sex, (digital) health literacy, and distance to the hospital may increasingly play a significant role in satisfaction and efficacy outcomes. As healthcare becomes more centralized to improve patient outcomes and lower costs, patients may face longer travel distances, which particularly impacts socioeconomically disadvantaged groups and the elderly, who may encounter greater challenges in accessing care. OVC could provide a valuable solution to these challenges, ensuring accessibility. Future studies should incorporate these considerations. Sixth, none of the included RCTs specifically addressed patient and surgeon satisfaction in the high-demand pre-surgical consultation or evaluated quality in terms of patient information recall. Since follow-up visits are less demanding and potentially more suitable for OVC, future studies should specifically address this aspect.

## Conclusion

This systematic review identified limited and heterogeneous evidence from 14 studies (including seven RCTs) on the use of OVC in patient–surgeon consultation in terms of patient and surgeon satisfaction and efficacy. Randomized trials specifically targeting the role of OVC in the pre-surgical consultation are lacking and no study has compared the impact of OVC versus F2F consultation on patient knowledge recall (i.e. the main purpose of the consultation). Also, only few studies [4, 22] addressed the potential increased use of F2F appointments in patients after OVC. Future randomized controlled trials should investigate these aspects, to confidently allow the implementation of OVC in care trajectories at surgical outpatient clinics.

## Supplementary Information

Below is the link to the electronic supplementary material.

Supplementary file1 (DOCX 33 kb)
