# Supplementary material for: Online video versus face-to-face patient–surgeon consultation: a systematic review
Source: Surg Endosc. 2024 Nov 5;38(12):7064–72. doi: 10.1007/s00464-024-11307-7 (PMC11614914; doi:10.1007/s00464-024-11307-7)
Supplement: Supplementary file 1 — Supplementary file1 (DOCX 33 kb) [file 464_2024_11307_MOESM1_ESM.docx]

**Supplementary Materials - Index**

| **Supplementary Methods** |  |
| --- | --- |
| Search | *pag. 2* |
| **Supplementary Tables** |  |
| Table S1. bias assessment randomized controlled trials | *pag. 3* |
| Table S2. bias assessment non-randomized controlled trials | *pag. 4* |
|  |  |

**Supplementary Methods**

Ovid/Medline Results (4 December 2023)

| **Search** | **Ovid/Medline Query – Dec 4, 2023** | **Results** |
| --- | --- | --- |
| #3 | exp Surgical Procedures, Operative/ or (surger* or surgical or operation or preoperati* or presurger* or operative).ti,ab,kf. | 4967871 |
| #2 | (videoconsult* or (video adj3 consult*)).ti,ab,kf. | 1890 |
| #1 | 1 and 2 | 567 |

Embase.com Results (4 December 2023)

| **Search** | **Embase Query – Dec 4, 2023** | **Results** |
| --- | --- | --- |
| #4 | #3 NOT 'conference abstract'/it | 277 |
| #3 | #1 AND #2 | 409 |
| #2 | 'surgery'/exp OR surger*:ti,ab,kw OR surgical:ti,ab,kw OR operation:ti,ab,kw OR preoperati*:ti,ab,kw OR presurger*:ti,ab,kw OR operative:ti,ab,kw | 6864013 |
| #1 | 'video consultation'/exp OR ((video NEAR/3 consult*):ti,ab,kw) OR videoconsult*:ti,ab,kw | 2544 |

Clarivate Analytics/Web of Science Core Collection Results (4 December 2023)

| **Search** | **Web of Science Query – Dec 4, 2023** | **Results** |
| --- | --- | --- |
| #1 | TS=((video NEAR/3 consult*) OR videoconsult*) | 1744 |
| #2 | TS=(surger* OR surgical OR operation OR preoperati* OR presurger* OR operative) | 3326939 |
| #3 | #1 AND #2 | 177 |

**Supplementary Tables**

| **Table S1. Bias assessment randomized controlled trials** | | | | | | |
| --- | --- | --- | --- | --- | --- | --- |
| **Author, Year** | **Domain 1: Risk of bias arising from the randomization process** | **Domain 2: Risk of bias due to deviations from the intended interventions** | **Domain 3: Risk of bias due to missing outcome data** | **Domain 4: Risk of bias in measurement of the outcome** | **Domain 5: Risk of bias in selection of reported result** | **Overall risk-of-bias judgement** |
| **Westra, 2015** | Some Concerns | Some Concerns | Some Concerns | Some Concerns | Some Concerns | **Some Concerns** |
| **Viers, 2015** | Low Risk | Low Risk | Some Concerns | Some Concerns | Some Concerns | **Some Concerns** |
| **Buvik, 2016** | Low Risk | Some Concerns | Low Risk | Some Concerns | Some Concerns | **Some Concerns** |
| **Damery, 2021** | Low Risk | High Risk | Some Concerns | Some Concerns | Some Concerns | **High Risk** |
| **Lee, 2021** | Low Risk | Low Risk | Low Risk | Some Concerns | Some Concerns | **Some Concerns** |
| **Muschol, 2022** | Low Risk | Some Concerns | Some Concerns | Low Risk | Some Concerns | **Some Concerns** |
| **Sada, 2023** | Some Concerns | High Risk | Some Concerns | Some Concers | Some Concerns | **High Risk** |
| Risk of bias assessment using the Cochrane Risk-of-Bias Tool 2 for randomized trials. | | | | | | |

| **Table S2. Bias assessment non-randomized controlled trials** | | | | | | | | | |
| --- | --- | --- | --- | --- | --- | --- | --- | --- | --- |
| **Author, Year** | **Study design** | **Bias due to confounding** | **Bias in selection of participants into the study** | **Bias of classification of interventions** | **Bias due to deviations from intended intervention** | **Bias due to missing data** | **Bias in measurement of outcomes** | **Bias in selection of the reported outcomes** | **Overall risk-of-bias judgement** |
| **Sellars, 2020** | PC | Serious Risk | Serious Risk | Serious Risk | Moderate Risk | Moderate Risk | Moderate Risk | Moderate Risk | **Serious Risk** |
| **Barsom, 2021** | PC | Serious Risk | Moderate Risk | Low Risk | Moderate Risk | Low Risk | Moderate Risk | Moderate Risk | **Serious Risk** |
| **Schumm, 2021** | PC | Serious Risk | Moderate Risk | Low Risk | Moderate Risk | Moderate Risk | Moderate Risk | Moderate Risk | **Serious Risk** |
| **Sharma, 2021** | RC | Serious Risk | Critical Risk | Serious Risk | Moderate Risk | Serious Risk | Moderate Risk | Moderate Risk | **Critical Risk** |
| **Sibanda, 2021** | RC | Serious Risk | Serious Risk | Serious Risk | Moderate Risk | Serious Risk | Moderate Risk | Serious Risk | **Serious Risk** |
| **Mahmoud, 2022** | MC | Moderate Risk | Serious Risk | Moderate Risk | Moderate Risk | Serious Risk | Moderate Risk | Moderate Risk | **Serious Risk** |
| **Baxter, 2023** | RC | Moderate Risk | Moderate Risk | Serious Risk | Moderate Risk | Moderate Risk | Moderate Risk | Moderate Risk | **Serious Risk** |
| Risk of bias assessment using the ROBINS-I Tool for non-randomized controlled trials. PC = prospective cohort study, RC = retrospective cohort study, MC = mixed (prospective and retrospective) cohort study. | | | | | | | | | |
